# Vi-Vaccinations Induce Heterogeneous Plasma Cell Responses That Associate With Protection From Typhoid Fever

**DOI:** 10.3389/fimmu.2020.574057

**Published:** 2020-12-03

**Authors:** Deborah L. Cross, Marije K. Verheul, Michael D. Leipold, Gerlinde Obermoser, Celina Jin, Elizabeth Jones, Joshua S. Starr, Irina Mohorianu, Christoph J. Blohmke, Holden T. Maecker, Giorgio Napolitani, Jennifer Hill, Andrew J. Pollard

**Affiliations:** ^1^ The Oxford Vaccine Group, Department of Paediatrics, University of Oxford, and the NIHR Oxford Biomedical Research Centre, Oxford, United Kingdom; ^2^ The Human Immune Monitoring Center, Institute for Immunity, Transplantation and Infection, Stanford School of Medicine, Stanford, CA, United States; ^3^ MRC Human Immunology Unit, MRC Weatherall Institute of Molecular Medicine, University of Oxford, Oxford, United Kingdom

**Keywords:** Salmonella Typhi, Vi-conjugate vaccine, typhoid fever, mass cytometry, CyTOF, ELISpot assay

## Abstract

Vi-polysaccharide conjugate vaccines are efficacious against cases of typhoid fever; however, an absolute correlate of protection is not established. In this study, we investigated the leukocyte response to a Vi-tetanus toxoid conjugate vaccine (Vi-TT) in comparison with a plain polysaccharide vaccine (Vi-PS) in healthy adults subsequently challenged with *Salmonella* Typhi. Immunological responses and their association with challenge outcome was assessed by mass cytometry and Vi-ELISpot assay. Immunization induced significant expansion of plasma cells in both vaccines with modest T follicular helper cell responses detectable after Vi-TT only. The Vi-specific IgG and IgM B cell response was considerably greater in magnitude in Vi-TT recipients. Intriguingly, a significant increase in a subset of IgA^+^ plasma cells expressing mucosal migratory markers α4β7 and CCR10 was observed in both vaccine groups, suggesting a gut-tropic, mucosal response is induced by Vi-vaccination. The total plasma cell response was significantly associated with protection against typhoid fever in Vi-TT vaccinees but not Vi-PS. IgA^+^ plasma cells were not significantly associated with protection for either vaccine, although a trend is seen for Vi-PS. Conversely, the IgA^-^ fraction of the plasma cell response was only associated with protection in Vi-TT. In summary, these data indicate that a phenotypically heterogeneous response including both gut-homing and systemic antibody secreting cells may be critical for protection induced by Vi-TT vaccination.

## Introduction

Vi polysaccharide (Vi-PS) vaccines targeting *Salmonella enterica* serovar Typhi (*S*. Typhi) are moderately protective against typhoid fever but are not widely used in endemic countries. Immunological memory is poorly induced by plain polysaccharide antigens, compromising their long-term efficacy (1). Glycoconjugate vaccines, in which polysaccharide antigens are chemically conjugated to a carrier protein, elicit superior antibody titres in infants (2, 3) in a T-cell-dependent immune response that leads to germinal center formation and antibody maturation (4). In addition, conjugate vaccines do not induce hypo-responsiveness to subsequent doses in the way that plain polysaccharide vaccines do, allowing for boosting regimens to be implemented (5). These mechanisms have been exploited with great success in vaccines targeting encapsulated bacteria, including those against *Streptococcus pneumoniae* and *Neisseria meningitidis*, which has resulted in the inclusion of these glycoconjugate vaccines in routine infant immunization schedules (6, 7). A recently developed Vi-tetanus toxoid (Vi-TT) conjugate vaccine was shown to be protective in a controlled human challenge model of typhoid fever (8) and demonstrated an efficacy of 81.6% in a Phase III, randomized, controlled trial in Nepal (9). Immunogenicity of Vi-TT has been found to be significantly higher in comparison with Vi-PS (10).

Production of adequate quantities of functional systemic antibody forms only one component of a protective immune response. Migration of antibody secreting cells to effector sites may be important for localized antibody production. Antibody-mediated recognition of *Salmonella* pathogens may be a key mechanism for driving antigen presentation to T-cells and for cellular cytotoxicity *via* Fc binding (11). Mucosal immunity is also thought to be a factor in protection against a number of enteric infections including cholera, rotavirus and typhoid fever (12–14). Chemotaxis of effector cells throughout the body relies on surface expression of homing receptors (15). Tissue specific homing to the small intestine is primarily mediated by alpha 4 beta 7 integrin (α4β7) and C-C chemokine receptor 9 (CCR9), while C-C chemokine receptor 10 (CCR10) mediates trafficking of cells to both the small and large intestine. Parenteral administration of vaccines is considered a poor method for inducing mucosal immune responses (16–18). However, a number of studies refute this paradigm (19–23). Currently, there are no detailed studies describing the gut homing response to Vi parenteral vaccines and how these cell types correlate with protection from *S*. Typhi infection.

Here, the cellular response to immunization was assessed in healthy volunteers subsequently challenged with *Salmonella* Typhi. Magnitude and homing potential of the plasma cell response were assessed and their association with protection from typhoid fever is described.

## Materials and Methods

### Study Design

Samples for this work were obtained from a randomized, controlled, phase 2b clinical trial centered in Oxford, UK evaluating the efficacy of Vi-TT in deliberately infected volunteers (ClinicalTrials.gov identifier: NCT02324751). Details of the study protocol and inclusion criteria were published previously (8). Healthy adults received a Vi-tetanus toxoid conjugate vaccine (Vi-TT: Typbar-TCV, Bharat Biotech), or a Vi plain polysaccharide vaccine (Vi-PS: TYPHIM Vi, Sanofi Pasteur). Participants were monitored in an outpatient setting and serial blood samples were collected at baseline (D0), and 7 (D7), 10 (D10), and 28 (D28) days post-vaccination. Participants were challenged orally approximately 28 days post-vaccination with 1–5 x 10^4^ colony forming units (CFU) of *S*. Typhi Quailes strain. In context of the current manuscript, all samples were collected before the participants were challenged. Informed written consent was obtained from all volunteers before enrolment.

### Mass Cytometry

Cryopreserved peripheral blood mononuclear cell (PBMC) samples were selected based upon sample availability for deep immune phenotyping using mass cytometry (or Cytometry by Time-of-Flight, CyTOF). Serial samples from 39 volunteers (Vi-PS n=20, Vi-TT n=19), collected on at baseline (D0), 7 (D7), and 28 days (D28) post-vaccination were selected; participant characteristics are described in **Supplementary Table 1**. An antibody panel targeting 37 surface-expressed cellular markers was used for phenotyping and allowed identification of all major PBMC subsets (see **Supplementary Table 2** for details of the panel). Staining and data acquisition was performed at the Human Immune Monitoring Centre (HIMC, Stanford University, USA).

Samples were prepared for mass cytometry and run in batches. PBMCs were thawed in a water bath at 37°C in RPMI+ benzonase washes. Cell viability was assessed by Vi-Cell (Beckman Coulter). Mass-tag barcoding of each sample was conducted using a combination of two palladium isotope-tagged CD45 antibodies diluted in CyFACS (20 mM potassium phosphate, 150 mM NaCl, 0.1% BSA, 2mM EDTA pH 8.0) per sample to enable subsequent multiplexing (24). All barcoded PBMC samples from a single study participant and a single sample from a healthy control donor were then pooled prior to further processing. Pooled samples were incubated with a primary antibody panel diluted in CyFACS at room temperature for 30 min (see **Supplementary Table 2**, all antibodies either Fluidigm or produced in-house). Samples were washed three times with CyFACS buffer prior to incubation with a secondary antibody panel diluted in CyFACS at room temperature for 30 min. Cells were washed three times with CyFACS then stained at room temperature with cisplatin (Fluidigm, USA) to confirm viability and fixed with 2% paraformaldehyde. Nucleated cells were stained with an iridium intercalator (Fluidigm, USA) for 20 min at room temperature. Finally, cells were washed three times (once with CyFACS, twice with MilliQ water) and resuspended in MilliQ water with EQ lanthanide-embedded polystyrene normalization beads to ~0.7 M cells/ml for acquisition on a mass cytometer. Data were acquired on Helios instruments (Fluidigm, USA). A target of acquisition of 250,000 events. Ungated events per sample resulted in a range of 20,000–200,000 live intact singlets per sample (.fcs files stored in Flow Repository Accession ID: FR-FCM-Z2GG).

The Premessa R package (https://github.com/ParkerICI/premessa) was used with the EQ normalization beads to account for variance in machine performance (25). Each.fcs file containing barcoded samples was deconvoluted to separate events from individual samples and successful deconvolution was confirmed in FlowJo. Marker expression was arcsinh-transformed prior to clustering and data visualization. Gating of major cell populations was performed for the healthy control sample run in each of the pools. Data from the healthy control samples were not taken further in the analysis, but can be found in Flow Repository (Repository ID: FR-FCM-Z2GG).

### Data Analysis

Identification of cell sub-populations was carried out using both clustering and manual gating strategies. A total of 100,000 live intact singlets were subsampled with replacement from each.fcs file to obtain a balanced input for both clustering and manual gating. To ensure representative subsampling, a median expression intensity within 10% of original sample median for each marker and a correlation of 0.9999 between expression values in the original files and the subsampled files was deemed satisfactory (**Supplementary Figure 3**).

Clustering analysis (using Kohonnen self-organizing maps) was performed using the R package FlowSOM (26). Expression of all markers were used to define clusters. Data were centered (z-score standardization) and logicle-transformed before clustering based on Euclidean distance. Multiple seeds were tested to ensure the reproducibility of outputs.

Manual gating was performed in FlowJo Version 10.0 using subsampled files and focused on CD38^++^ B-cells and T follicular helper (Tfh) cells (see **Supplementary Figure 4** for the gating strategies used). Statistical differences between time points were calculated using Wilcoxon matched-pairs signed rank tests or Mann-Whitney U tests, as appropriate.

### ELISpot Assays

Antigen-specific antibody secreting cells (ASC) and memory B-cell responses were assessed using enzyme-linked immunosorbent spot (ELISpot) assays. **Supplementary Figure 5** shows overlap with the samples that were part of the CyTOF analysis.

***Ex-Vivo* ELISpot:** ASC responses to Vi, lipopolysaccharide (LPS) and tetanus toxoid (TT) were evaluated at baseline, 7, 10, and 28 days post-vaccination. Briefly, 96-well multiscreen filter plates (Merck Millipore, Burlington, USA) pre-coated with antigen (Vi-polysaccharide 12/244, Lot 2039, NIBSC, Potters Bar, UK, coating concentration 10 g/ml; *S*. Typhi LPS, Lot 072K4082 Sigma L2387, Dorset UK, coating concentration 10 g/ml; tetanus toxoid, Lot T177-2 and T224-01, Statens Serum Institut, Copenhagen, Denmark, coating concentration 5 g/ml) were loaded with 2.5 x 10^5^ PBMCs (four wells per sample), and incubated at 37°C with 5% CO_2_ overnight. Wells pre-coated with pan goat anti-human immunoglobulin (H17000, Caltag, Buckingham, UK, coating concentration 10 g/ml) and PBS served as positive and negative controls, respectively. Plates were developed using alkaline phosphatase-conjugated secondary antibodies (Calbiochem/Merck, Burlington, USA) and substrate development kits (Bio-Rad Laboratories Ltd, Watford, UK). Plates were counted using an AID ELR03 ELISpot reader (Autoimmun Diagnostika, Germany). Data are presented as the number of ASCs per million PBMCs, calculated by subtracting the mean of the PBS wells from the mean of antigen-containing wells and adjusting for starting PBMC count. The lower limit of detection was 3 per 10^6^ cells for Vi-specific IgG and IgM ASCs and was 6 per 10^6^ cells for tetanus-specific ASCs. Upper limits of detection were assigned for wells with excessive spots (141 per 10^6^ cells for Vi-specific IgG ASCs, 348 per 10^6^ cells for Vi-specific IgM ASCs, 529 per 10^6^ cells for tetanus-specific ASCs). Samples that did not generate spots in pan-IgG/M-coated wells were excluded from the analysis.

**Memory B-Cell ELISpot:** Memory ELISpots were conducted using cryopreserved PBMCs. Samples were thawed, washed, and resuspended at a concentration of 2x10^5^ cells per well in complete media (RPMI supplemented with 10% heat inactivated fetal bovine serum, 1% L-glutamine, 1% penicillin/streptomycin, 1% MEM Non-essential amino acids, 1% sodium pyruvate, 0.1% b-mercaptoethanol). 100 μl of antigen mix, composed of *Staphylococcus aureus* Cowans strain (VWR International Ltd, 1/5000 dilution), CpG-ODN 2006 (tlrl-2006-5 Invivogen, 1.7 g/ml) and pokeweed mitogen (L-9379 Sigma, 83.33ng/ml) was added to each well. Plates were cultured for 5 days at 37°C, 5% CO_2,_ and 95% humidity to stimulate plasmablast differentiation from memory B-cells. Cells were then harvested and washed and plates were developed using the same method described above to detected Vi-specific IgG and tetanus-specific IgG ASCs. The upper limit of detection was 300 per 10^6^ cells for Vi-specific IgG ASCs and 500 per 10^6^ for tetanus-specific IgG ASCs.

### Serum Vi-IgA Quantification

Serum Vi IgA antibodies were quantified using an adapted protocol based on the VaccZyme Human Anti-S typhi Vi IgG ELISA kit (VaccZyme, Birmingham, UK). The secondary antibody was replaced with goat anti human IgA prepared 1:12000 in 1 x phosphate buffered saline and 10% fetal bovine serum.

## Results

### Vi-Specific ASCs Are Induced by Both Vi-TT and Vi-PS Vaccines While Memory B-Cell Responses Are Detected Only After Vi-TT Vaccination

**Figure 1:**
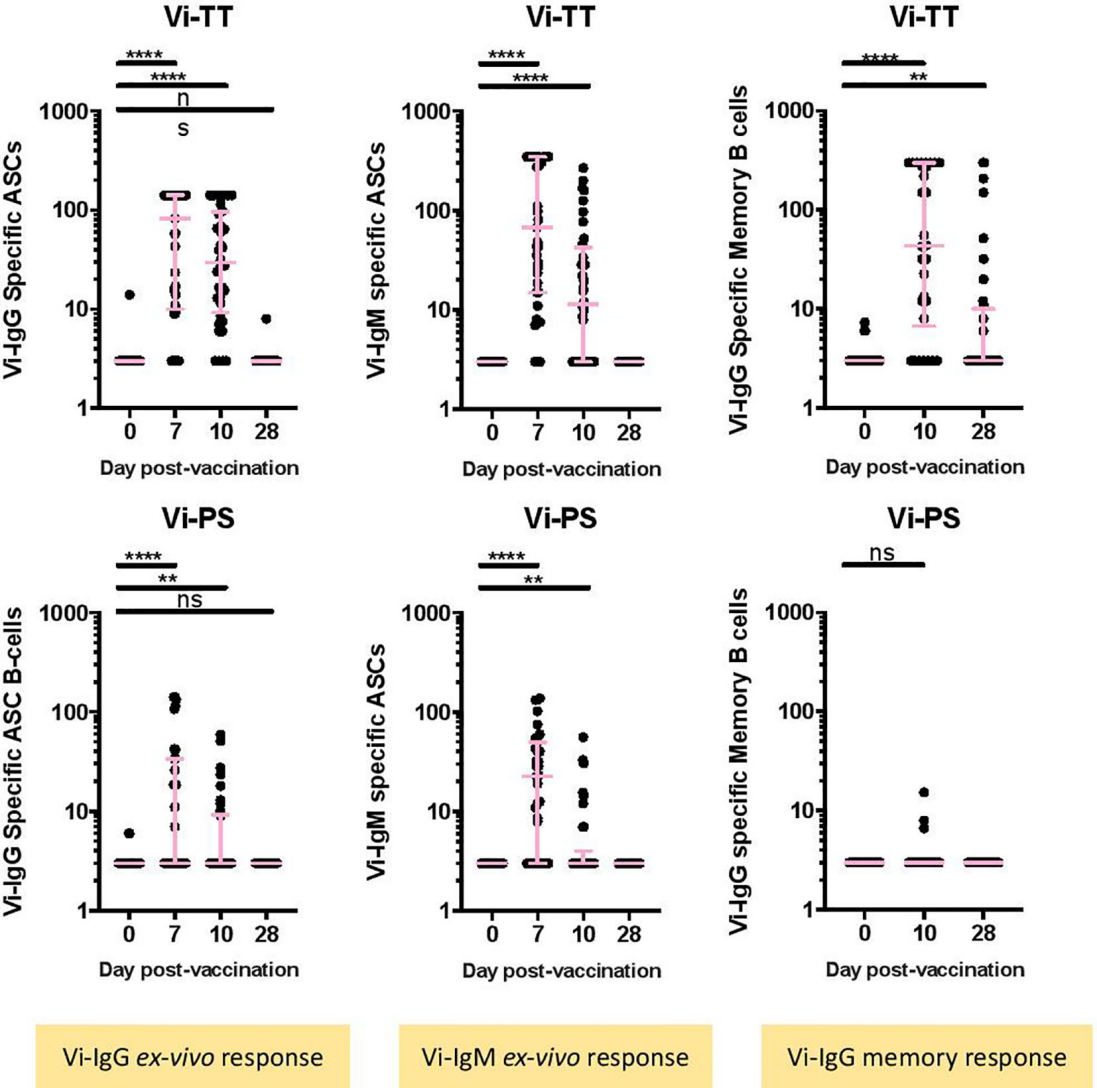
Vi-specific plasmablasts and memory B cells are increased after conjugate or polysaccharide vaccination. **(A)** The number of IgG or IgM Vi-specific antibody secreting cells (ASC) **(B)** or memory B cells expressed as specific cell / 10^6^ cells was measured by ELISpot. Results for recipients of the Vi-TT vaccine are shown in the top row and for recipients of Vi-PS in the bottom row. Significance was determined by a Wilcoxons signed paired rank test (two-tailed). **p < 0.01, ****p < 0.0001, ns, not significant. In some cases, there were no differences in ranks between two comparisons, in this case no results are indicated in the figure. The mean frequency for each group and 95% confidence intervals are plotted. D0, Day 0, day of vaccination; D7, 7 days after vaccination; D28, 28 days after vaccination; CyTOF, Cytometry Time of Flight; Vi-PS, Vi-polysaccharide vaccine; Vi-TT, Vi-tetanus conjugate vaccine; TT, Tetanus.Group sizes: *Ex-vivo* ASC Vi-TT: n = 39 *Ex-vivo* ASC Vi-PS: n = 35 Memory B-cells, Vi-TT: n = 30 Memory B-cells, Vi-PS: n = 29.

The numbers of Vi-specific IgG and IgM antibody secreting cells (ASCs) were determined by ELISpot following vaccination. Vi-specific IgG ASCs were detected in peripheral blood 7 days post-vaccination in Vi-PS and Vi-TT vaccinees (**Figure 1A**, Vi-PS: n = 35, Vi-TT: n = 39, **Supplementary Figure 1** for individual participant ASC kinetics). Significantly higher frequencies of Vi-specific IgG ASCs were detected in Vi-TT vaccinees in comparison with Vi-PS; median 82.5 per 10^6^ PBMCs (IQR: 10-141) versus 3 per 10^6^ PBMCs (IQR 3-33.5) for Vi-TT and Vi-PS, respectively. Vi-specific IgG ASCs were also detected at 10 days following vaccination, however the frequency of ASCs detected was lower than 7 days post-vaccination for both groups (Vi-PS: n = 34, Vi-TT: n = 36). Vi specific IgM-expressing ASCs followed the same pattern as for IgG in both vaccine groups. The frequency of tetanus-specific IgG ASCs increased 7 days following vaccination in Vi-TT vaccinees only, median 301 per 10^6^ PBMCs (IQR: 188-529) (**Supplementary Figure 6**, Vi-PS: n = 34, Vi-TT: n = 30).

A significant increase in Vi-specific IgG memory B-cells was detected at 10 and 28 days following Vi-TT vaccination. No significant changes in Vi-specific IgG memory B-cells were observed in Vi-PS vaccinees (**Figure 1B**). Ten days after vaccination, 23/30 (76.7%) Vi-TT vaccinees had Vi-specific IgG memory B-cells detectable in peripheral blood in comparison with 3/29 (10.3%) for the Vi-PS vaccinees. Vi-specific IgG memory B-cells were still detectable 28 days following vaccination in 11/36 (30.6%) of Vi-TT vaccinees while no responses were detectable in 34 Vi-PS vaccinees. No LPS-specific memory cell response was detected (**Supplementary Figure 6**). Combined, these data quantify the substantial differences in Vi-specific immunity following vaccination with Vi-TT in comparison with Vi-PS. Differences are present for all B cell isotypes evaluated. In addition, we provide evidence for the successful induction of Vi-specific memory responses after Vi-TT immunization. As expected, Vi-PS was a poor inducer of systemic memory, in keeping with other literature on plain polysaccharide vaccines.

### Vi-TT Vaccination Induces Gut-Homing CD38^++^ B-Cells

**Figure 2:**
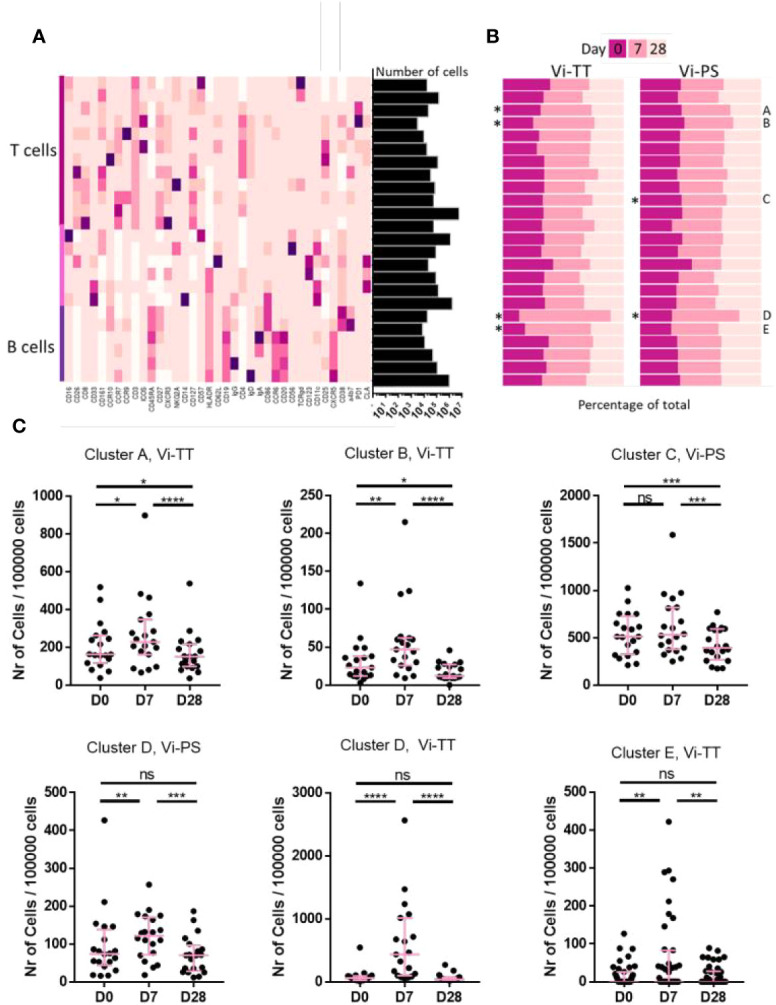
Cellular responses to vaccination are mostly present after conjugate vaccination. **(A)** A broad analysis was carried out using all available cellular markers for hierarchical clustering in FlowSOM. A total of 24 clusters was generated. The heatmap shows the median expression of a cellular marker for each of the clusters. A darker color indicates a higher expression of that particular marker. Expression level visualization was scaled by column. Clusters were designated as T cells, B cells or other cells based on manual inspection of marker expression and grouped by cell type. The total number of cells in each of the clusters is indicated in the bars on the right hand side. The total number of cells is 11,700,000. **(B)** The contribution of cells from a particular time point to each cluster is shown split by vaccine arm. As the number of cells for each of the timepoints is similar, bars are expected to be equal (at 33%) if the number of cells is equal between timepoints. Differences in cell frequency between time points were calculated using a Related-samples Friedman’s Two-way Analysis of Variance by Ranks. Significant differences are indicated by "*". Significantly different clusters were labelled A-E for further referencing. **(C)** Each dot represents the number of cells from one individual at a specific timepoint within the indicated cluster. Only clusters that were significantly changed in **(B)** are shown. Significance was determined by a Wilcoxons signed paired rank test. *p < 0.05, **p < 0.01, ***p< 0.001, ****p < 0.0001, ns, not significant. The mean frequency for each group and 95% confidence intervals are plotted. D0, Day 0, day of vaccination; D7, 7 days after vaccination; D28, 28 days after vaccination; CyTOF, Cytometry Time of Flight; Vi-PS, Vi-polysaccharide vaccine; Vi-TT, Vi-tetanus conjugate vaccine.

We sought to further investigate the characteristics of plasma cells and other cellular subsets induced by Vi vaccinations. In an expansive phenotyping study, the expression of 37 markers across a range of PBMC subsets was measured. Clustering based on expression intensity was performed to derive cell types. Changes in frequency of different subsets was then assessed. When applied to our dataset, the FlowSOM algorithm derived 24 clusters (**Figure 2A**) (see **Supplementary Figure 7** for a more detailed overview) five of which (two B-cell and three T-cell clusters) were significantly changed following vaccination (**Figure 2B**). In Vi-PS recipients, there was a marked expansion of a B-cell cluster characterized by expression of typical plasma cell markers including CD19, CD27, and CD38, 7 days post-vaccination (**Figure 2C**, cluster D, p = 0.041, D0 vs D7, n = 20). The same cluster was also expanded in Vi-TT vaccinees (p = <0.0001, D0 vs D7, n = 19). An additional, smaller plasma cell cluster was also observed to only change in Vi-TT recipients (**Figure 2C**, cluster E, p = 0.0016, D0 vs D7) characterized by CD19, CD38, and α4β7 expression with intermediate levels of CCR10.

### Both Vi Vaccines Alter CD38^++^ B-Cell Homing and Activation

**Figure 3:**
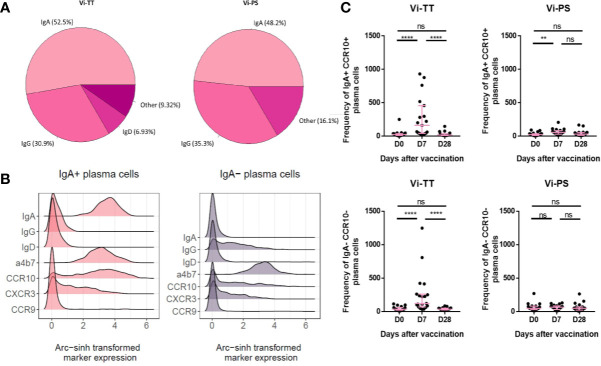
Vi immunization induces a sub-population of gut-homing B-cells. **(A)** Surface immunoglobulin expression across plasma cells from either Vi-TT or Vi-PS vaccinees was assessed by manual gating at 7 days post-vaccination. **(B)** Cells from clusters D and E were grouped according to their expression of IgA. Homing marker expression on IgA+ and IgA- cells is shown using density plots. **(C)** The frequencies of IgA+ and IgA- plasma cells at baseline, 7, and 28 days after vaccination with either Vi-TT (n = 19) or Vi-PS (n = 20). Each dot represents the number of plasma cells from an individual at a specific time point. Significance was determined by a Wilcoxons signed paired rank test (two-tailed). **p < 0.01, ****p < 0.0001, ns, not significant. Participants in **(B)** were vaccinated with Vi-TT and participants in **(C)** were vaccinated with Vi-PS. Mean frequency for each group and 95% confidence intervals are plotted. D0, Day 0, day of vaccination; D7, 7 days after vaccination; D28, 28 days after vaccination; CyTOF, Cytometry Time of Flight; Vi-PS, Vi-polysaccharide vaccine; Vi-TT, Vi-tetanus conjugate vaccine.

The expanded CD38^++^ plasma cell clusters were investigated further by manual gating in FlowJo to examine homing marker and surface immunoglobulin expression. Interestingly, we observed that a considerable proportion of plasma cells induced by both vaccines at day 7 expressed IgA (**Figure 3A**, Vi-PS: n = 20, Vi-TT: n = 19). When we further investigated the phenotype of IgA^+^ plasma cells, we observed they had a distinctive phenotype and kinetics after vaccination in comparison with IgA^-^ plasma cells. IgA^+^ plasma cells expressed integrin α4β7 and a significant subset of these co-expressed the chemokine receptors CCR10 and CXCR3 (**Figure 3B**). Integrin α4β7 and CCR10 co-expression is characteristic of a gut-directed plasma cell response and mediates migration to both the large and small intestine. Notably, none of the IgA^+^ subsets of plasma cells expressed CCR9, the chemokine receptor mediating migration to the small intestine (27). IgA^-^ plasma cells also expressed α4β7 but were otherwise generally lower in expression of all homing markers investigated (**Figure 3B**). The frequency of IgA^+^ plasma cells strongly increased after vaccination, particularly in the Vi-TT group. IgA^-^ plasma cells were significantly changed in Vi-TT vaccinees only (**Figure 3C**).

**Figure 4:**
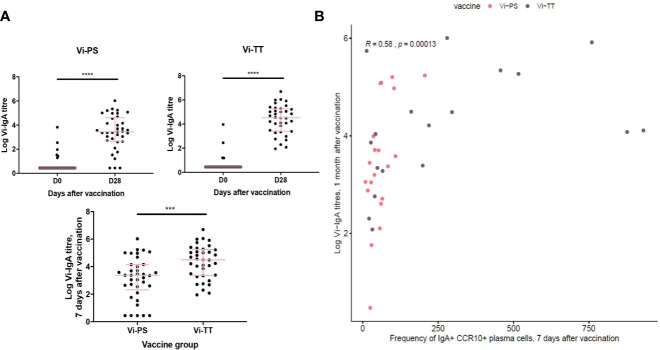
Gut-tropic plasma cells correlated with serum titres of Vi-IgA. **(A)** Serum Vi-IgA titres were measured pre- and post-vaccination. Titres are represented on a Log2 scale. Each dot represents one participant At baseline in the Vi-TT group, n = 33. At 7 days post-vaccination, n = 36. At baseline in the Vi-PS group, n = 37. At seven days post-vaccination, n = 37. Significance was determined via a Wilcoxons signed paired rank test (two-tailed). ***p< 0.001, ****p < 0.0001, ns, not significant. **(B)** The frequency of CCR10+ IgA+ plasma cells 7 days after vaccination were correlated with logged Vi-IgA titres 1 month after vaccination using a Spearman’s correlation. In the Vi-PS group, n = 20. In the Vi-TT group, n = 19.

To address the lack of information about antigen specificity in our phenotyping data, we correlated the frequency of IgA^+^ α4β7^+^ CCR10^+^ plasma cells with Vi-IgA titres measured at 1 month after vaccination (Vi-TT: n = 33, Vi-PS: n = 37). Serum levels of Vi-IgA were significantly increased after both immunizations; this increase was greatest in Vi-TT recipients (**Figure 4A**). The frequency of IgA^+^ α4β7^+^ CCR10^+^ plasma cells 7 days after immunization correlated with antibody titre, supporting the assertion that a proportion of IgA^+^ cells are Vi-specific (**Figure 4B**).

These results imply that the induced CD38^++^ plasma cell response following vaccination is highly heterogeneous in terms of isotype and homing pattern. Interestingly, these data also indicate that a significant proportion of the response to these parenteral immunizations express a distinctively mucosal phenotype which correlates with antigen-specific humoral immunity.

### Vi-TT Vaccination Induces Changes in Circulating Tfh Cells

Other cell subsets observed to significantly change in frequency between time points are of the T-cell lineage (**Figure 2C**, clusters A–C). The frequency of cells present in cluster C detected 28 days following Vi-PS vaccination, was significantly reduced when compared with frequencies detected at baseline and D7. No significant change in cluster C was observed between D0 and D7. This cluster was highly heterogeneous and consisted of multiple T-cell populations as evidenced by expression of CD3, CD4, CD8, CD127, and CCR7. The T-cell independent nature of Vi-PS vaccination and the kinetics of the change in cluster C suggests it is unlikely to represent a response to vaccination. Vaccine-mediated T-cell responses are generally found to occur much earlier than 1 month (28). Therefore, this cluster has not been investigated in greater detail. The frequency of cells in cluster A, which consist of both CD8^+^ and CD4^+^ T-cells expressing PD-1, significantly increased 7 days after vaccination in the Vi-TT group. Twenty-eight days after vaccination, the frequency of cluster A cells was significantly lower compared with baseline. A similar pattern was observed in Vi-TT vaccinees for a second T-cell cluster, cluster B, which consisted of ICOS and PD-1 positive CD4 T-cells. Cluster B was further characterized by expression of T-cell activation markers (CD25, CD27, CD38) and lymph node homing markers including CCR7 and CD62L. However, T-cell clusters that significantly changed following Vi-TT vaccination did not strongly associate with Vi-specific humoral responses (data not shown).

**Figure 5:**
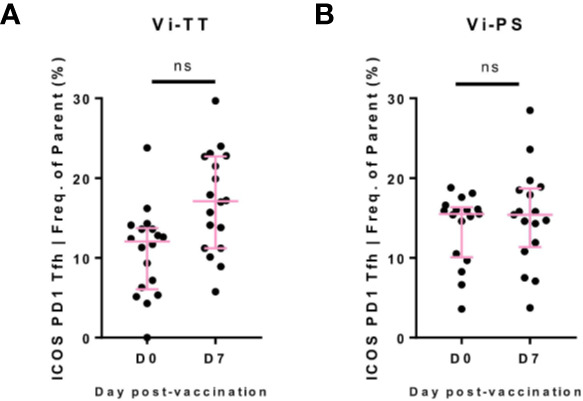
Circulating Tfh after Vi vaccination. **(A)** Circulating T-follicular helper cells (cTfh)were identified by manual gating as CD3+CD4+CXCR5+PD-1+ICOS+(see supplementary figure 3 for representative example of gating strategy). For each participant, numbers of cTfh are plotted as a frequency of the CD4+CXCR5+population in Vi-TT recipients. **(B)** And Vi-PS recipients. Significance for all was determined by a Wilcoxons signed paired rank test (two-tailed). ns, not significant. With n = 35 for all, n = 18 for Vi-TT and n = 17 for Vi-PS. Data were excluded from analysis if less than 20 cells in the final gate were obtained. D0, Day 0, day of vaccination; D7, 7 days after vaccination; CyTOF, Cytometry Time of Flight; Vi-PS, Vi-polysaccharide vaccine; Vi-TT, Vi-tetanus conjugate vaccine.

Given the T-cell dependent nature of the response to Vi-TT, we anticipated a significant rise in circulating Tfh cells post-vaccination. Whilst a single cluster of Tfh was not identified by the FlowSOM algorithm, a fraction of cells in cluster B expressed the canonical markers indicative of a Tfh phenotype (CD4, CXCR5, PD-1, ICOS) (data not shown). To further investigate the behavior of Tfh upon vaccination, we identified this population by manual gating (CD3^+^ CD4^+^ CXCR5^+^ CD45RA- PD-1^+^ ICOS^+^). A non-significant increase at D7 was present in Vi-TT vaccinees (**Figure 5**, Vi-TT: n = 18, Vi-PS: n = 17).

### Upon Vaccination With Vi-TT Changes in Cell Frequencies Within CD38^++^ B-Cell Clusters Are Associated With Protection Against Typhoid Fever

**Figure 6:**
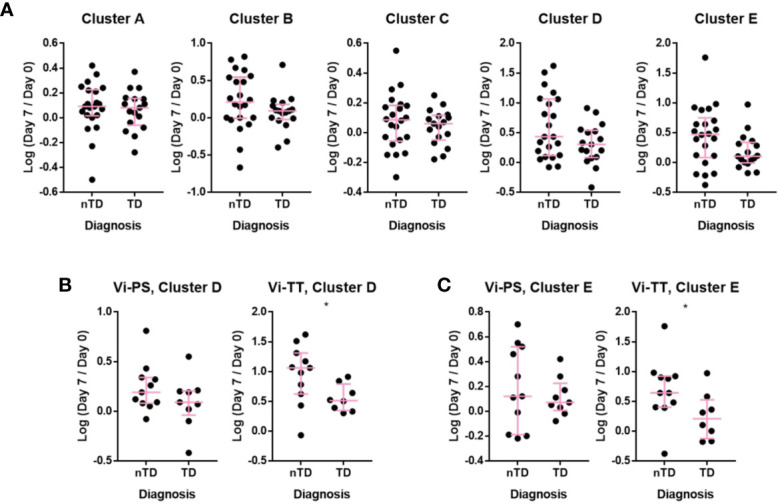
Individuals protected upon *S.* Typhi challenge had increased plasmablast changes after Vi-TT vaccination. The change in the number of cells in a cluster at D7 compared to D0 is shown as the log-transformed ratio between D7 and D0. Participants are split into those who developed typhoid and those who did not after challenge with *S.* Typhi. **(A)** Clusters A–E, showing participants from Vi-TT and Vi-PS groups combined. **(B)** Change in the number of cells in Cluster D with recipients of Vi-PS and Vi-TT plotted separately **(C)** change in the number of cells in Cluster E with recipients of Vi-PS and Vi-TT plotted separately. For the Vi-TT group: protected participants (nTD), n = 11, diagnosed participants (TD), n = 8. For the Vi-PS group, protected participants (nTD), n = 11, diagnosed participants (TD), n = 9. Significance was determined by a Mann-Whitney test. *p < 0.05. D0, Day 0, day of vaccination; D7, 7 days after vaccination; Vi-PS, Vi-polysaccharide vaccination; Vi-TT, Vi-tetanus conjugate vaccine, nTD, not diagnosed with typhoid fever, TD, diagnosed with typhoid fever.

**Figure 7:**
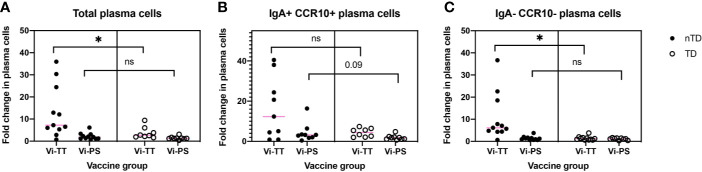
Vi-vaccine plasma cell responses have differential associations with protection from typhoid fever. The association between protection from disease and **(A)** the total plasma cell response (Vi-TT, n = 19, Vi-PS, n = 20) **(B)** the IgA+ CCR10+ plasma cell response (Vi-TT, n = 17, Vi-PS, n = 20) or **(C)** the IgA- CCR10- plasma cell response (Vi-TT, n = 19, Vi-PS, n = 20) is shown for both vaccine groups. Significance was determined by a Mann-Whitney test. *p < 0.05. D0, Day 0, day of vaccination; D7, 7 days after vaccination; Vi-PS, Vi-polysaccharide vaccine; Vi-TT, Vi-tetanus conjugate vaccine, nTD, not diagnosed with typhoid fever, TD, diagnosed with typhoid fever, TD = diagnosed with typhoid fever.

The association between observed changes in cellular frequency and protection from typhoid fever after challenge with live bacteria at D28 were investigated. When combining individuals from both vaccine arms, protected participants generally exhibited a greater change in all of the clusters A–E following vaccination when compared with participants diagnosed with typhoid fever (**Figure 6A**). When the vaccine arms were evaluated separately, a greater fold change in CD38^++^ plasma cell clusters D and E was significantly associated with protection from disease in Vi-TT (**Figures 6B, C**, n = 19), but not Vi-PS (n = 20). Plasma cell responses induced by both vaccines were further investigated in detail for their association with protection against typhoid fever. The fold rise of the gated total plasma cell response (irrespective of tissue tropism or immunoglobulin expression) was strongly associated with protection in Vi-TT vaccinees (p = 0.026) but not Vi-PS (p = 0.201). (**Figure 7A**). In recipients of Vi-TT strong induction of plasma cells without markers of mucosal tropism (IgA- CCR10-) was found to be protective (p = 0.033) whereas generation of IgA^+^ CCR10^+^ -expressing plasma cells was not associated with protection (p = 0.277) (**Figure 7B**). In contrast, induction of non-mucosal plasma cells was not significantly different between outcome groups amongst recipients of Vi-PS (p = 0.761) whereas mucosal plasma cells had a stronger relationship with protection (p = 0.094) (**Figure 7C**). These findings suggest that that Vi-vaccinations may mediate protection *via* different mechanisms with both systemic and mucosal responses potentially playing a role.

## Discussion

This is the first study to describe in detail and compare cellular responses following vaccination with a Vi-conjugate or plain Vi-polysaccharide vaccine. We have identified a heterogeneous plasma cell signature following vaccination that associates with protection from typhoid fever after experimental challenge. In keeping with previous studies of Vi-PS, we have identified a predominance of IgA^+^ CD38^++^ plasma cells 7 days post-vaccination with relatively lower induction of IgG^+^ plasma cells (29). Vi-TT was also found to induce potent proliferation of IgA^+^ plasma cells, but with a stronger induction of IgG responses than Vi-PS as is expected for a conjugate vaccine. Consistent with studies of other conjugate and polysaccharide vaccines (30, 31), the overall magnitude of the plasma cell response is greater for Vi-TT than Vi-PS. These findings further elucidate the cellular responses that follow these immunizations (32).

An interesting observation in our study is that a subset of IgA^+^ plasma cells detectable after both Vi-immunizations co-express the mucosal homing markers α4β7 and CCR10. Activated plasma cells are conferred with tissue-specific homing markers at the site of initial antigen encounter (33, 34). The chemokine receptor CCR10 is a pan-mucosal migration marker and induces homing of numerous leukocyte subsets to sites such as the gut, mammary gland, salivary gland and trachea (35, 36). Co-expression with the surface integrin, α4β7, is well-documented in mediating migration of IgA^+^ plasma cells to the gastrointestinal tract (37). High endothelial venules (HEVs) of the gut-associated lymphoid tissue are distinct from other mucosal tissues for their high expression of the α4β7 ligand, MadCAM-1, that facilitates cell extravasation from blood into the tissues (38–40). Induction of gut homing effector cells may be a key feature of host defenses against enteric pathogens. In murine models of *S*. Typhimurium infection, several cell types have been found to localize to the small intestine, including monocytes, effector T-cells and plasmablasts (41–43). indicating that migration to the mucosal surface is a crucial aspect of host immunity. Induction of α4β7-positive plasmablasts against other enteric pathogens such as *Shigella* is also thought to be important in protection (44).

The detection of gut-tropic plasma cells was unexpected after administration of two parenteral vaccines. These findings could imply that a proportion of the IgA^+^ plasma cells observed after vaccination are derived from a memory B-cell population previously primed at the mucosal surface. Mucosally activated B-cells have been shown to contribute to the memory pool in bone marrow and spleen (45, 46). The clonal relatedness of serum IgA antibodies and intestinal plasma cells in patients with celiac disease further suggests that mucosal responses are contributing to immunity beyond the gut (47). Vi ELISpots found no evidence of memory cells in peripheral blood, although Vi-IgA ELISpots were not carried out. Participants with history of exposure to *Salmonella* Typhi and/or previous Vi vaccination were excluded from our study as much as possible. Vi expression has been demonstrated on other *Enterobacteriaceae* species such as *Citrobacter freundii*, a commensal pathogen typically responsible for nosocomial infections associated with immunocompromised patients (48–50). The prevalence of C. *freundii* carriage in the UK adult population and in our cohort is unknown. Given the systemic nature of Vi-vaccination, it is unclear whether prior mucosal exposure would plausibly have an effect on plasma cell responses to vaccination. The possibility of Vi-specific memory B-cells contributing to a secondary lymphoid niche has not been investigated here.

We observe an association between plasma cell responses to vaccination and protection from disease after challenge in Vi-TT participants only. Previously, our lab has found that Vi-IgG antibody titres after vaccination in Vi-TT recipients, whilst high, were unable to distinguish protected and diagnosed individuals (8). Here, we find that greater induction of IgA^-^ CCR10^-^ plasma cells was significantly associated with protection in Vi-TT participants. IgA^+^ CCR10^+^ plasma cells were increased in protected participants of both vaccine arms albeit not significantly. This trend was strongest in Vi-PS vaccinees. This may suggest that both magnitude and localization of the plasma cell response are important factors in protection from typhoid fever. Other immune responses beyond plasma cells that also play a role in protection have not been factored into these comparisons.

Beyond B-cells, other cellular compartments were largely unchanged, or exhibited only modest changes at the time points measured. This may reflect a lack of substantial responses to Vi-PS/Vi-TT vaccination by other cell types, and/or kinetics of other populations (e.g. innate cells) that differ from those of B-cells. Whilst a rise in Tfh cells was observed in the Vi-TT group after vaccination, it did not reach statistical significance. Most previous studies detailing changes in Tfhs in peripheral blood have examined attenuated viral vaccines, known to induce strong T-cell dependent responses, although some responses after glyco-conjugate vaccination have been described (51–53). Notably, these studies also did not find correlations between Tfh frequencies and polysaccharide antibody titres which is in-keeping with our findings. It is likely that the relatively low cell numbers analyzed hindered our capacity to detect significant differences in Tfh cell frequencies, and that power to detect small differences was limited by the sample size. Detection of memory B-cell responses to Vi following immunization does however indicate productive germinal center responses are induced by Vi-TT as anticipated.

To conclude, this study has identified populations of mucosally-directed and systemic plasma cells induced after two parenteral Vi-vaccinations that differentially associate with outcome of infection. Vi-PS appears to mediate some of its protective effects at the mucosa whereas Vi-TT seems to rely on induction of a high magnitude, systemic response. The application of CyTOF to vaccine evaluation has been little exploited but has the potential to offer a deeper understanding of responses that associate with protection from disease. To thoroughly explore our data both clustering and gating strategies were used to provide the most robust conclusions. These findings contribute to our increasing understanding of vaccine-mediated protection from typhoid fever.

## Data Availability Statement

The datasets presented in this study can be found in online repositories. The names of the repository/repositories and accession number(s) can be found below: Flow Repository—FR-FCM-Z2GG.

## Ethics Statement

The studies involving human participants were reviewed and approved by South Central Oxford A Ethics Committee (14/SC/1427). The patients/participants provided their written informed consent to participate in this study.

## Author Contributions

The study design was conceived by CB, CJ, and JH with the oversight of AP. The CyTOF data were generated by ML at the Stanford Immune Monitoring Center under the coordination of HM and GO. ELISpot data were generated at the Oxford Vaccine Group by CJ, EJ, and JS. All analysis was conducted by DC and MV with guidance on bioinformatic methods provided by IM. DC and MV wrote the manuscript with feedback from all authors. All authors contributed to the article and approved the submitted version.

## Funding

Funding of the CyTOF study was provided by the Bill and Melinda Gates Foundation (OPP1113682) *via* the Global Health Vaccine Accelerator Platform (GH-VAP). Partial funding was provided by an instrument grant (S10RR027582) from the U.S. National Institutes of Health. The human challenge study was funded by The Bill & Melinda Gates Foundation (OPP1084259) and the European Commission FP7 grant “Advanced Immunization Technologies” (ADITEC) and supported by the NIHR Oxford Biomedical Research Centre.

## Conflict of Interest

AP is a National Institute for Health Research (NIHR) Senior Investigator, chairs the UK Department of Health and Social Care Joint Committee on Vaccination and Immunisation (JCVI) and is a member of the World Health Organization’s Strategic Advisory Group of Experts (SAGE). The views expressed in this article are those of the author(s) and not necessarily those of the NIHR, the Department of Health or the WHO.

The remaining authors declare that the research was conducted in the absence of any commercial or financial relationships that could be construed as a potential conflict of interest.
