# Supplementary material for: Vi-Vaccinations Induce Heterogeneous Plasma Cell Responses That Associate With Protection From Typhoid Fever
Source: Front Immunol. 2020 Dec 3;11:574057. doi: 10.3389/fimmu.2020.574057 (PMC7793947; doi:10.3389/fimmu.2020.574057)
Supplement: Supplementary file 1 [file DataSheet_1.pdf]

		Median age (IQR - in years)	Sex (Female)
CyTOF	Vi-TT	31.2 (22.6 - 50.1)	11/19 (58%)
	Vi-PS	29.2 (22.2 - 35.6)	9/20 (45%)
ELISpot	Vi-TT	25.5 (23.0 - 37.8)	19/41 (46%)
	Vi-PS	30.5 (22.9 - 44.4	13/37 (35%)

Supplementary Data 1 – Participant characteristics
Summary statistics are provided based on participants with baseline CyTOF or any ELISpot data. Exact age of participant at D0 was used for calculations.

Vi-TT – Vi Tetanus Toxoid Conjugate Vaccine, Vi-PS – Vi polysaccharide vaccine, IQR – interquartile range, CyTOf – cytometry time of flight, ELISpot – Enzyme-linked immunosorbent spot
