# Supplementary material for: Vi-Vaccinations Induce Heterogeneous Plasma Cell Responses That Associate With Protection From Typhoid Fever
Source: Front Immunol. 2020 Dec 3;11:574057. doi: 10.3389/fimmu.2020.574057 (PMC7793947; doi:10.3389/fimmu.2020.574057)
Supplement: Supplementary file 2 [file DataSheet_2.pdf]

Cocktail	Metal	Metal source	Antibody	Antibody Clone	Reagent source	Reagent supplier
Barcoding	89Y	Fluidigm	CD45	HI100	Commercial	Fluidigm
Barcoding	102Pd	Trace Sciences	CD45	HI100	in-house	Biolegend
Barcoding	105Pd	Trace Sciences	CD45	HI100	in-house	Biolegend
Barcoding	108Pd	Trace Sciences	CD45	HI100	in-house	Biolegend
Barcoding	110Pd	Trace Sciences	CD45	HI100	in-house	Biolegend
Primary	113In	Trace Sciences	CD57	HCD57	in-house	Biolegend
Primary	115In	Sigma-Aldrich	HLA-DR	G46.6	in-house	BD
Primary	141Pr	Fluidigm	CCR6	G034E3	Commercial	Fluidigm
Primary	142Nd	Fluidigm	CD19	HIB19	Commercial	Fluidigm
Primary	144Nd	Fluidigm	IgG	G18-145	in-house	BD
Primary	145Nd	Fluidigm	CD4	RPA-T4	Commercial	Fluidigm
Primary	146Nd	Fluidigm	IgD	IA6-2	Commercial	Fluidigm
Primary	147Sm	Fluidigm	CD20	2H7	Commercial	Fluidigm
Primary	148Nd	Fluidigm	IgA	G18-1	in-house	BD
Primary	149Sm	Fluidigm	CD56	NCAM16.2	Commercial	Fluidigm
Primary	150Nd	Fluidigm	CD86	IT2.2	Commercial	Fluidigm
Primary	151Eu	Fluidigm	ICOS	C398.4A	Commercial	Fluidigm
Primary	152Sm	Fluidigm	TCRgd	11F2	Commercial	Fluidigm
Primary	153Eu	Fluidigm	CD45RA	HI100	Commercial	Fluidigm
Primary	154Sm	Fluidigm	CD123	9F5	in-house	BD
Primary	155Gd	Fluidigm	CD27	L128	Commercial	Fluidigm
Primary	156Gd	Fluidigm	CXCR3	G025H7	Commercial	Fluidigm

Primary	NA	NA	NKG2A-PE	Z199	Commercial	Beckman-Coulter
Secondary	158Gd	Fluidigm	anti-PE	PE001	in-house	Biolegend
Primary	159Tb	Fluidigm	CD11c	Bu15	Commercial	Fluidigm
Primary	160Gd	Fluidigm	CD14	M5E2	Commercial	Fluidigm
Primary	161Dy	Fluidigm	CD26	BA5b	Commercial	Fluidigm
Primary	162Dy	Fluidigm	CD8a	RPA-T8	Commercial	Fluidigm
Primary	163Dy	Fluidigm	CD33	WM53	Commercial	Fluidigm
Primary	164Dy	Fluidigm	CD161	HP-3G10	Commercial	Fluidigm
Primary	165Ho	Fluidigm	CD127	A019D5	Commercial	Fluidigm
Primary	NA	NA	CCR10-APC	1B5	Commercial	BD
Secondary	166Er	Fluidigm	anti-APC	APC003	in-house	Biolegend
Primary	167Er	Fluidigm	CCR7	G043H7	Commercial	Fluidigm
Primary	168Er	Fluidigm	CCR9	L053E8	Commercial	Fluidigm
Primary	169Tm	Fluidigm	CD25	2A3	Commercial	Fluidigm
Primary	170Er	Fluidigm	CD3	UCHT1	Commercial	Fluidigm
Primary	171Yb	Fluidigm	CXCR5	RF8B2	Commercial	Fluidigm
Primary	172Yb	Fluidigm	CD38	HIT2	Commercial	Fluidigm
Primary	173Yb	Fluidigm	a4b7	Act1	in-house	NIH AIDS Reagent Program
Primary	174Yb	Fluidigm	PD-1	EH12.2H7	Commercial	Fluidigm
Primary	175Lu	Sigma-Aldrich	CD62L	DREG200	in-house	HIMC
Primary	176Yb	Fluidigm	CLA	HECA-452	Commercial	Fluidigm
Primary	209Bi	Fluidigm	CD16	3G8	Commercial	Fluidigm
Live-dead	Pt195	Fluidigm		cisplatin (nat. abund.)	Commercial	Fluidigm
Intercalator	Ir191	Fluidigm		Intercalator	Commercial	Fluidigm
Intercalator	Ir193	Fluidigm		Intercalator	Commercial	Fluidigm

Supplementary Data 2 – Overview of antibodies used in CyTOF staining
In-house conjugations performed by Dr. Michael Leipold at the Human Immune Monitoring Center (HIMC) at Stanford University
