# Supplementary material for: Vi-Vaccinations Induce Heterogeneous Plasma Cell Responses That Associate With Protection From Typhoid Fever
Source: Front Immunol. 2020 Dec 3;11:574057. doi: 10.3389/fimmu.2020.574057 (PMC7793947; doi:10.3389/fimmu.2020.574057)
Supplement: Supplementary file 3 [file DataSheet_3.pdf]

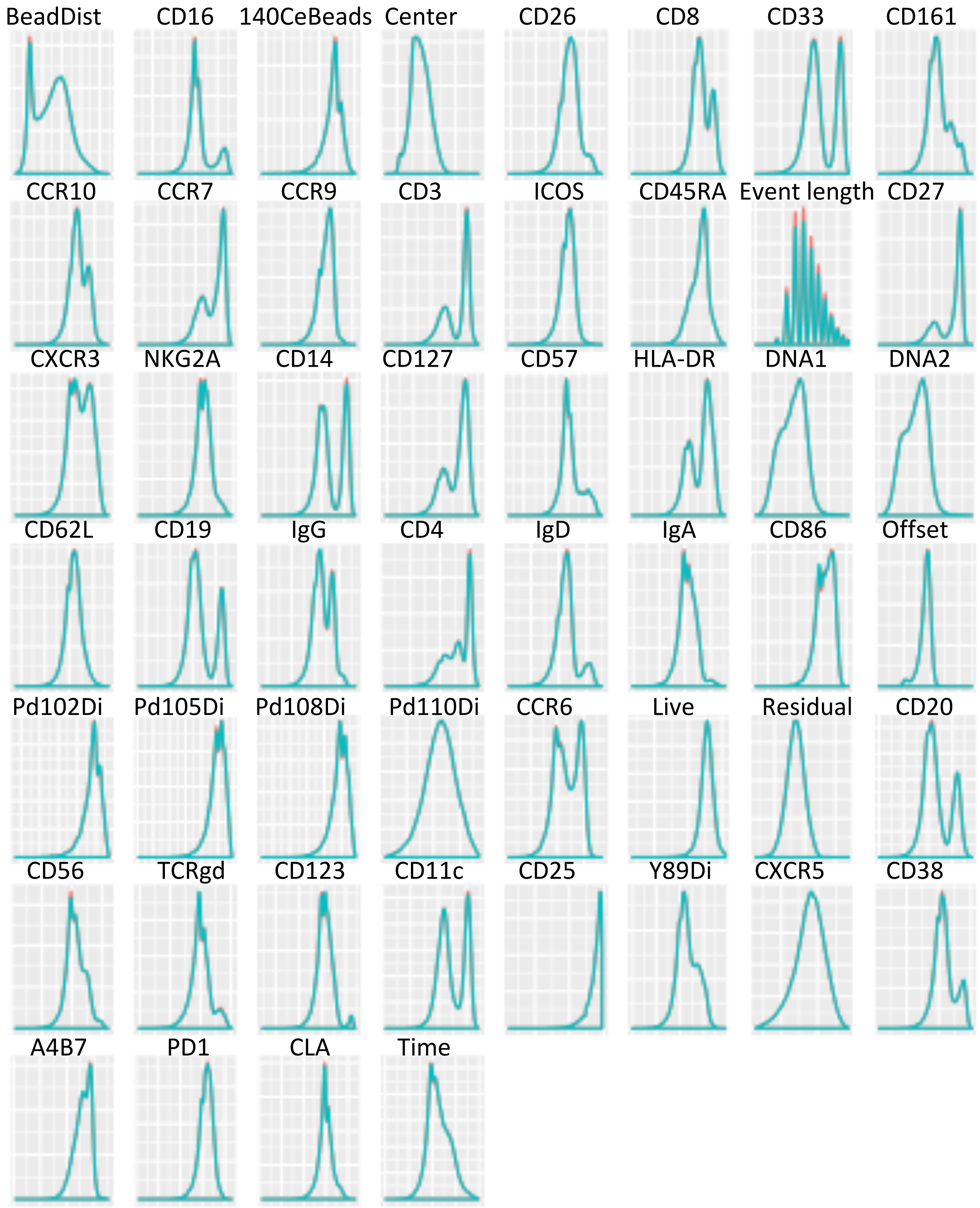

Supplementary Data 3 – Comparison of marker distribution between original and subsampled CyTOF data

The original distribution of marker (or variable) expression is indicated in red and for the subsampled data in blue for a representative sample. Strong overlap beween the distributions makes the the red color difficult to see in most cases. Only samples with no major differences in distribution as detected by eye, as well as meeting criteria for the difference in median expression intensity and correlation between expression values, were included.
