# Supplementary material for: Vi-Vaccinations Induce Heterogeneous Plasma Cell Responses That Associate With Protection From Typhoid Fever
Source: Front Immunol. 2020 Dec 3;11:574057. doi: 10.3389/fimmu.2020.574057 (PMC7793947; doi:10.3389/fimmu.2020.574057)

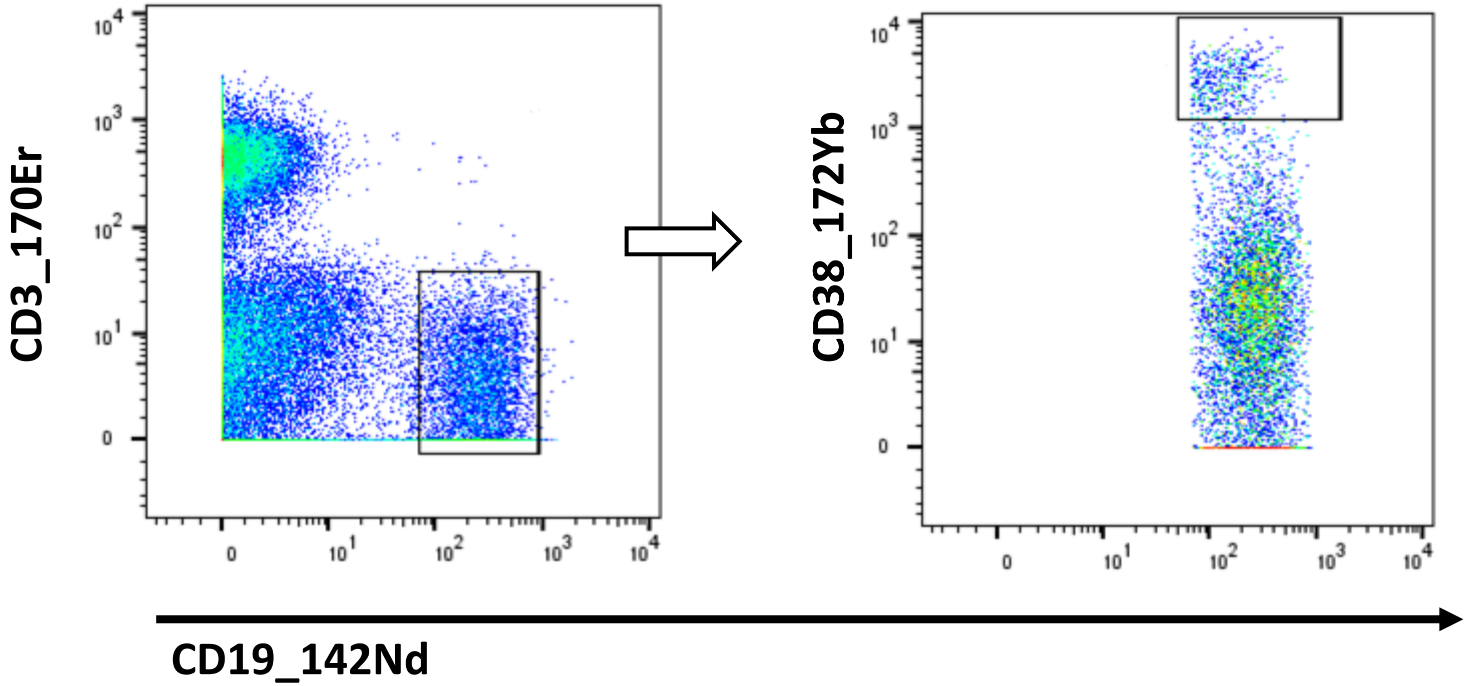

Supplementary Data 4 – Gating strategies

These figures show an overview of the two gating strategies that were used. The upper panel illustrates the gating of CD38++ plasma cells, while the lower panel illustrates the gating of PD1+ ICOS+ Tfh cells

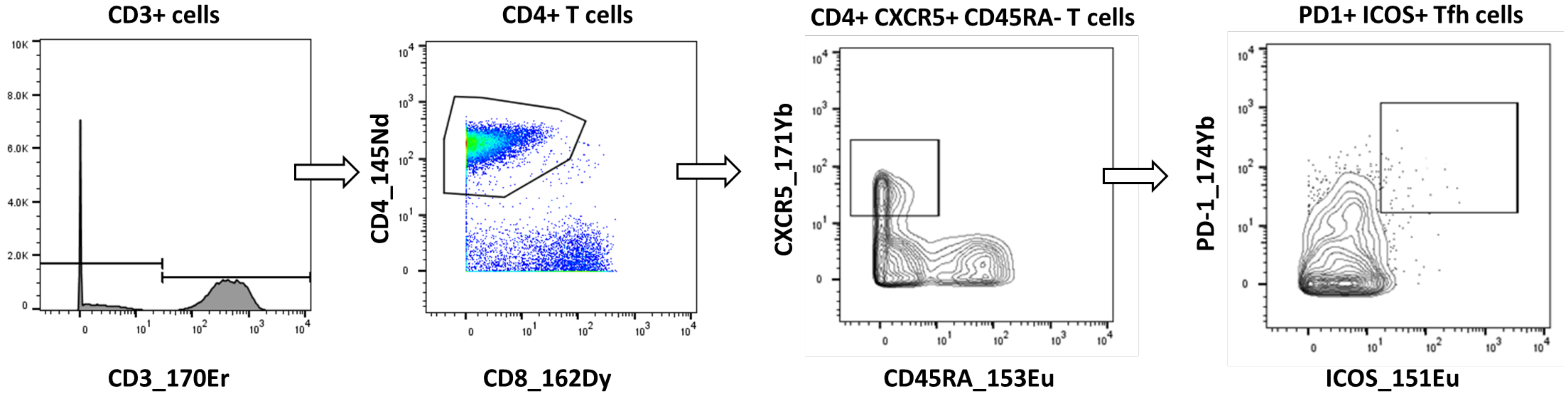

Supplement: Supplementary file 4 [file DataSheet_4.pdf]
