# Supplementary material for: Vi-Vaccinations Induce Heterogeneous Plasma Cell Responses That Associate With Protection From Typhoid Fever
Source: Front Immunol. 2020 Dec 3;11:574057. doi: 10.3389/fimmu.2020.574057 (PMC7793947; doi:10.3389/fimmu.2020.574057)
Supplement: Supplementary file 6 [file DataSheet_6.pdf]

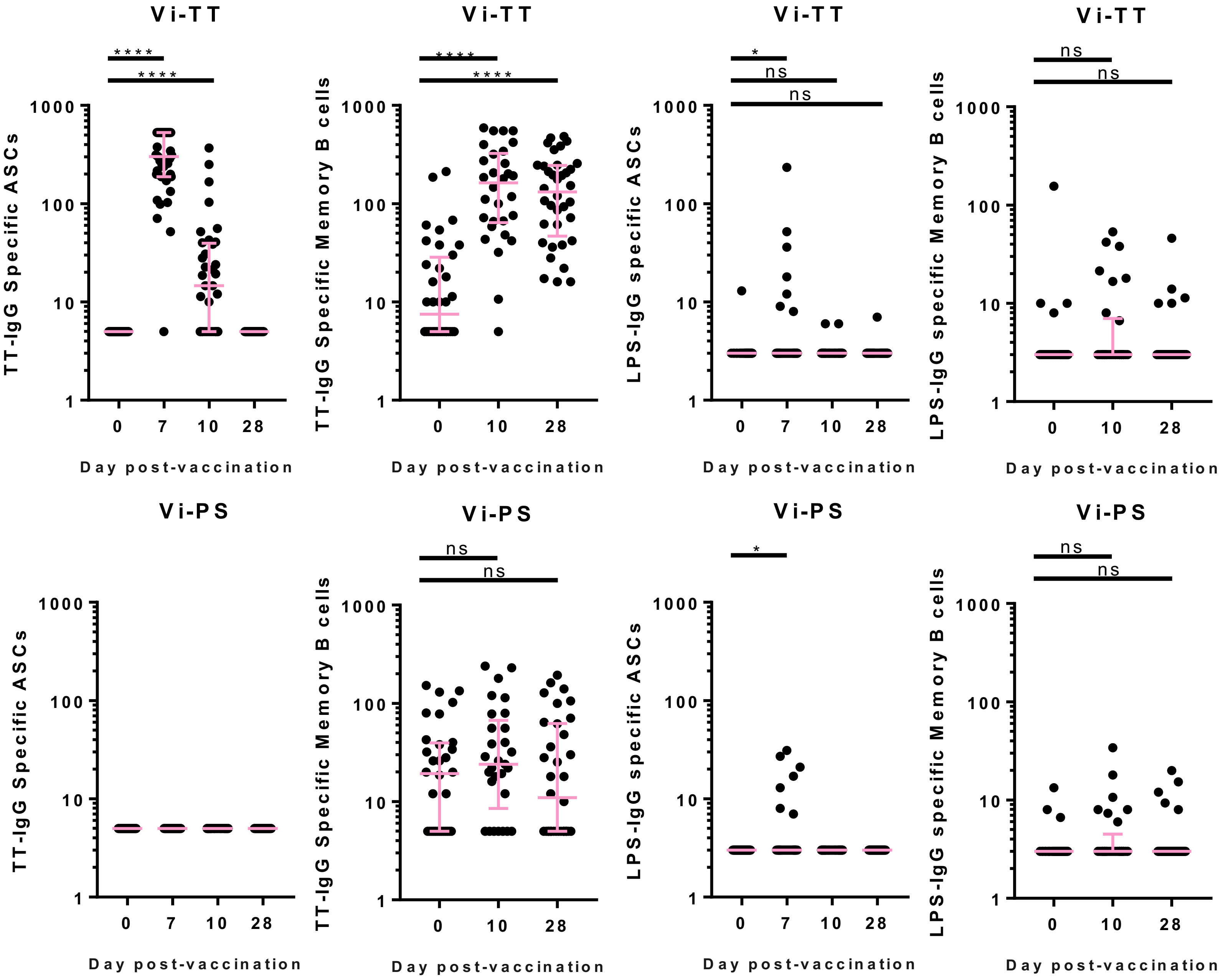

Supplementary Data 6 – TT-IgG and LPS ASC or memory B cells after conjugate or polysaccharide vaccination.
The number of TT-IgG or LPS-IgG specific antibody secreting cells (ASC) or memory B cells expressed as specific cell / 10^6 cells was measured by ELISpot. Results for recipients of the Vi-TT vaccine are shown in the top row and for recipients of Vi-PS in the bottom row.

Significance was determined by a wilcoxons signed paired rank test. * p <0.05, ** p <0.01, *** p<0.001, **** p <0.0001, ns = not significant.

Vi-PS = Vi-polysaccharide vaccine; Vi-TT = Vi-tetanus conjugate vaccine
