# Supplementary material for: Vi-Vaccinations Induce Heterogeneous Plasma Cell Responses That Associate With Protection From Typhoid Fever
Source: Front Immunol. 2020 Dec 3;11:574057. doi: 10.3389/fimmu.2020.574057 (PMC7793947; doi:10.3389/fimmu.2020.574057)
Supplement: Supplementary file 7 [file DataSheet_7.pdf]

Supplementary Data 7:

Archsinh-transformed marker intensity distributions for 37 markers across 24 clusters generated by FlowSOM. For convenience, clusters referenced throughout the paper were renamed A-E. In this plot, clusters are identifiable by their original cluster IDs (see below):

Cluster A: 18
Cluster B: 17
Cluster C: 16
Cluster D: 11
Cluster E: 8

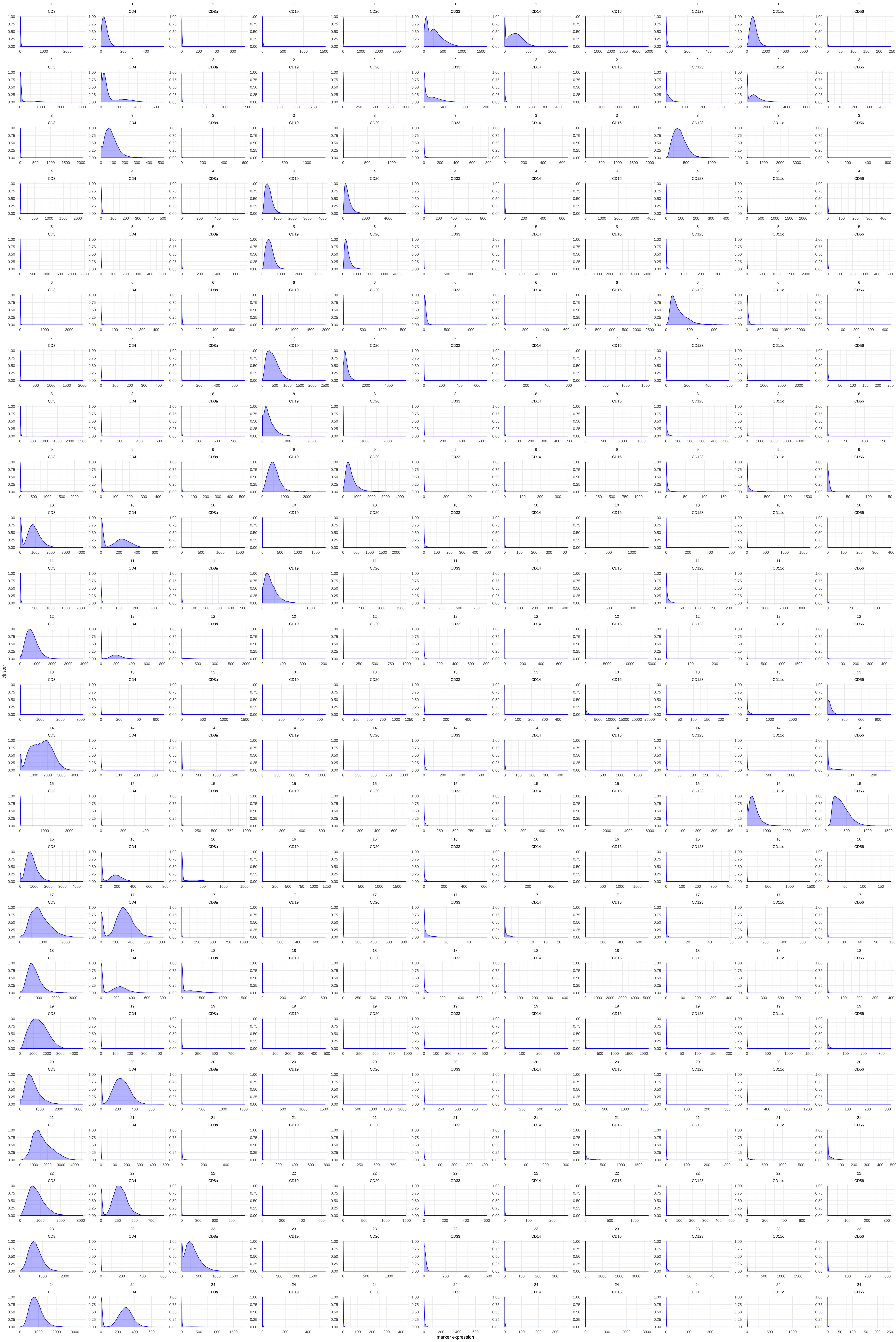
cluster
marker expression
CD3
CD4
CD8a
CD19
CD20
CD33
CD14
CD16
CD123
CD11c
CD56
1
2
3
4
5
6
7
8
9
10
11
12
13
14
15
16
17
18
19
20
21
22
23
24

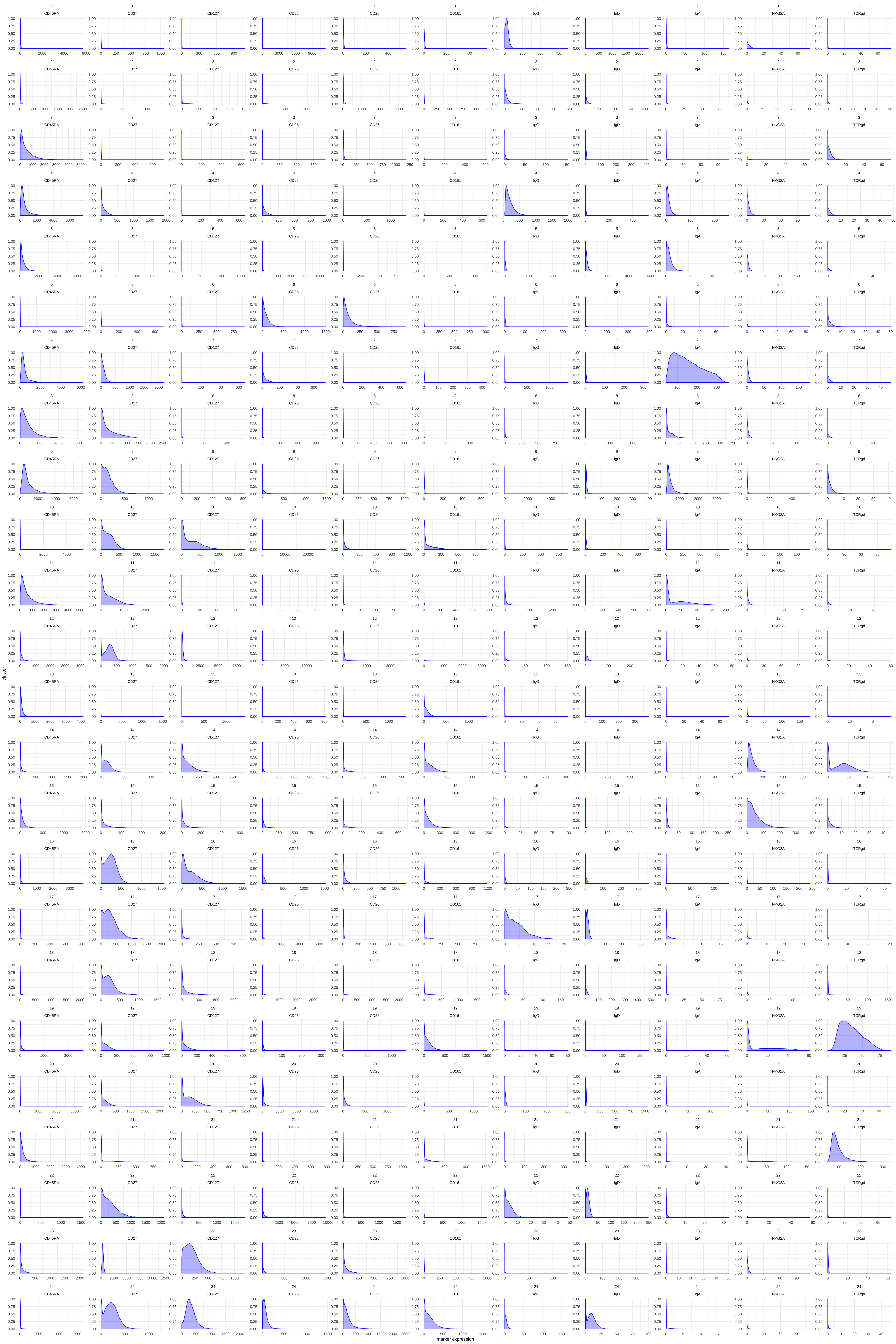
CD45RA
CD27
CD127
CD25
CD26
CD161
IgG
IgD
IgA
NKG2A
TCRgd
cluster
marker expression

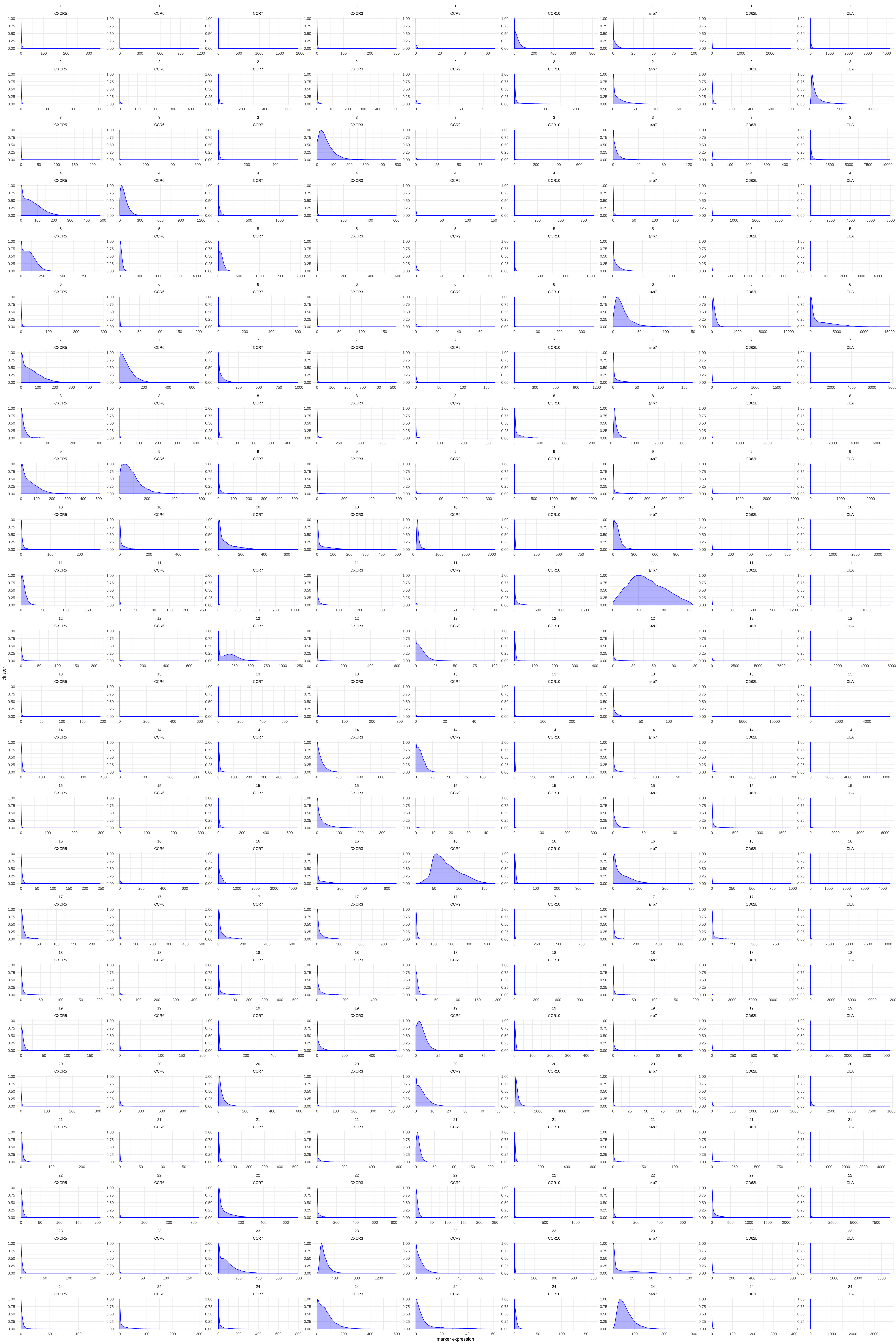
CXCR5
CCR6
CCR7
CXCR3
CCR9
CCR10
a4b7
CD62L
CLA
cluster
marker expression

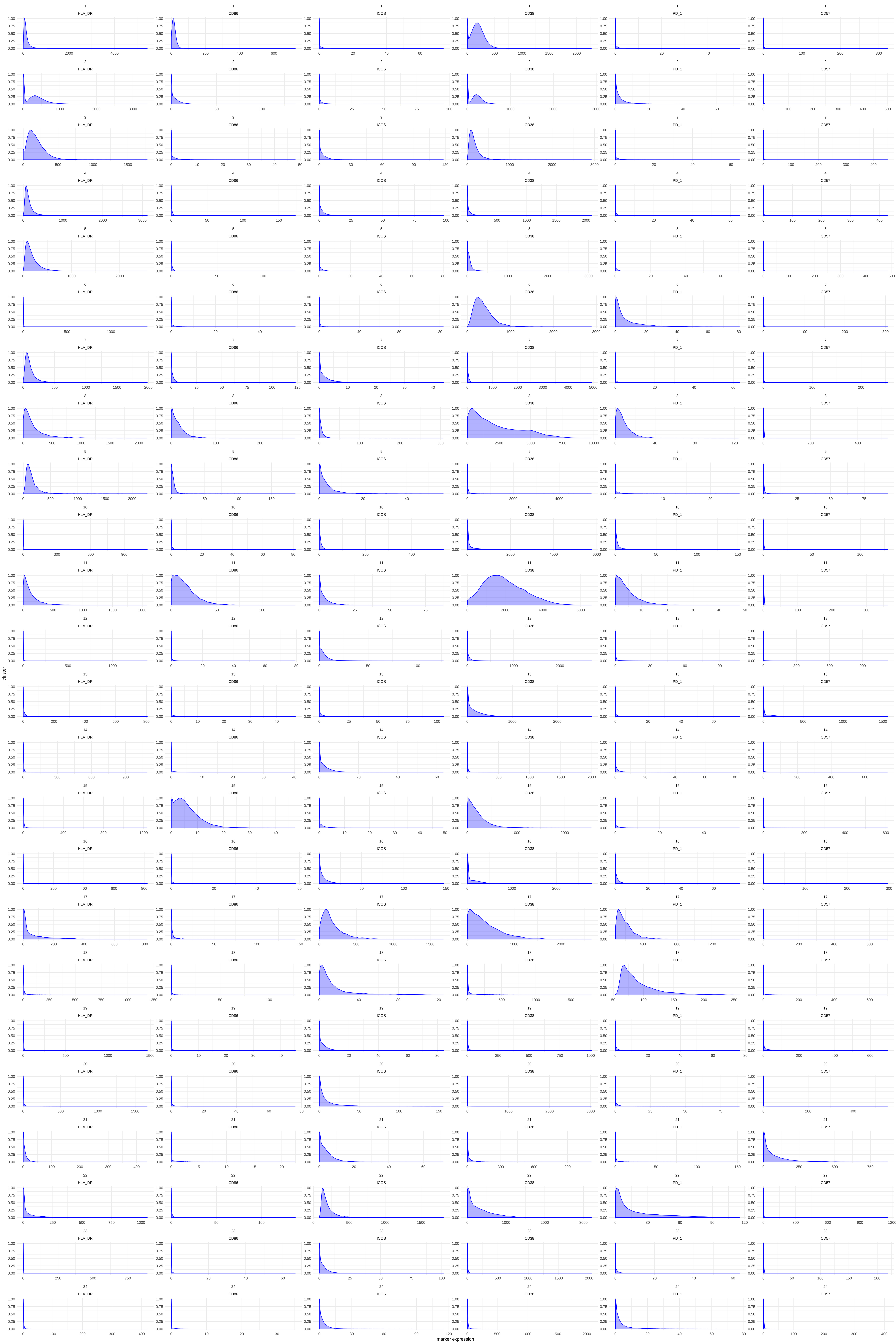

HLA_DR
CD86
ICOS
CD38
PD_1
CD57
cluster
marker expression
