# Supplementary material for: Vi-Vaccinations Induce Heterogeneous Plasma Cell Responses That Associate With Protection From Typhoid Fever
Source: Front Immunol. 2020 Dec 3;11:574057. doi: 10.3389/fimmu.2020.574057 (PMC7793947; doi:10.3389/fimmu.2020.574057)
Supplement: Supplementary file 5 [file DataSheet_5.pdf]

		D0		D7		D10	D28	
Vaccine	Challenge outcome	CyTOF	ELISpot	CyTOF	ELISpot	ELISpot	CyTOF	ELISpot
Vi-PS	NA							
Vi-PS	NA							
Vi-PS	nTD							
Vi-PS	nTD							
Vi-PS	nTD							
Vi-PS	nTD							
Vi-PS	nTD							
Vi-PS	nTD							
Vi-PS	nTD							
Vi-PS	nTD							
Vi-PS	nTD							
Vi-PS	nTD							
Vi-PS	nTD							
Vi-PS	nTD							
Vi-PS	nTD							
Vi-PS	nTD							
Vi-PS	nTD							
Vi-PS	nTD							
Vi-PS	nTD							
Vi-PS	nTD							
Vi-PS	nTD							
Vi-PS	nTD							
Vi-PS	nTD							
Vi-PS	nTD							
Vi-PS	TD							
Vi-PS	TD							
Vi-PS	TD							
Vi-PS	TD							
Vi-PS	TD							
Vi-PS	TD							
Vi-PS	TD							
Vi-PS	TD							
Vi-PS	TD							
Vi-PS	TD							
Vi-PS	TD							
Vi-PS	TD							
Vi-PS	TD							
Vi-TT	NA							
Vi-TT	NA							
Vi-TT	NA							
Vi-TT	NA							

Vi-TT	nTD							
Vi-TT	nTD							
Vi-TT	nTD							
Vi-TT	nTD							
Vi-TT	nTD							
Vi-TT	nTD							
Vi-TT	nTD							
Vi-TT	nTD							
Vi-TT	nTD							
Vi-TT	nTD							
Vi-TT	nTD							
Vi-TT	nTD							
Vi-TT	nTD							
Vi-TT	nTD							
Vi-TT	nTD							
Vi-TT	nTD							
Vi-TT	nTD							
Vi-TT	nTD							
Vi-TT	nTD							
Vi-TT	nTD							
Vi-TT	nTD							
Vi-TT	nTD							
Vi-TT	nTD							
Vi-TT	nTD							
Vi-TT	TD							
Vi-TT	TD							
Vi-TT	TD							
Vi-TT	TD							
Vi-TT	TD							
Vi-TT	TD							
Vi-TT	TD							
Vi-TT	TD							
Vi-TT	TD							
Vi-TT	TD							
Vi-TT	TD							
Vi-TT	TD							
Vi-TT	TD							

Supplementary Data 5 – Summary of participant samples included in the study. A coloured box indicates the use of a sample for a particular experiment. Empty boxes indicate that the sample was not included. The number of PBMCs available was the main factor involved in sample selection. An entry of 'NA' in the challenge outcome column indicates that a participant underwent vaccination but withdrew from the study prior to challenge with *S.* Typhi.

CyTOF
Memory ELISpot
ASC ELISpot
Both ELISpots

Vi-TT – Vi Tetanus Toxoid Conjugate Vaccine, Vi-PS – Vi-polysaccharide vaccine, nTD – no typhoid diagnosis, TD – typhoid diagnosis, CyTOF – cytometry time of flight, ELISpot – Enzyme-linked immunosorbent spot
